# Effects of cancer on patients with COVID-19: a systematic review and meta-analysis of 63,019 participants

**DOI:** 10.20892/j.issn.2095-3941.2020.0559

**Published:** 2021-02-15

**Authors:** Ludi Yang, Peiwei Chai, Jie Yu, Xianqun Fan

**Affiliations:** 1 Department of Ophthalmology; Shanghai Key Laboratory of Orbital Diseases and Ocular Oncology, Ninth People’s Hospital, Shanghai JiaoTong University School of Medicine, Shanghai 200025, China

**Keywords:** COVID-19, cancer, incidence, mortality, meta-analysis

## Abstract

**Objective:**

Patients with underlying diseases are more vulnerable to coronavirus disease 2019 (COVID-19). The purpose of this study was to investigate cancer incidence in patients with COVID-19 and to determine whether cancer was associated with mortality among patients with COVID-19.

**Methods:**

Electronic searches of PubMed, Embase, Cochrane, Web of Science, and medRxiv were conducted to collect studies that provided data regarding the incidence and mortality of cancer patients with COVID-19. Meta-analyses were used to estimate pooled incidences, risk ratios (RRs), and 95% confidence intervals (CIs) using a random-effects model. Heterogeneity among studies was detected using *I*^2^ statistics.

**Results:**

A total of 19 retrospective studies involving 63,019 patients (2,682 patients with cancer) were included. Meta-analysis showed that the pooled incidence of cancer in COVID-19 patients was 6% (95% CI: 3%–9%). The mortality rate of COVID-19 patients with cancer was higher than that of those without cancer [risk ratio (RR): 1.8, 95% CI: 1.38–2.35, *P* < 0.01]. Studies on specific types of cancer showed that among COVID-19 patients, the mortality rate of lung cancer patients was higher than that of patients without lung cancer (RR: 1.8, 95% CI: 0.85–3.80, *P* = 0.02).

**Conclusions:**

Patients with cancer were more susceptible to COVID-19. As a risk factor, cancer increased mortality among COVID-19 patients. Among COVID-19 patients with cancer, those who had lung cancer had a higher mortality than those without lung cancer. Our findings suggested that clinicians should pay more attention to cancer patients diagnosed with COVID-19 and provide useful information for their clinical management.

## Introduction

The novel coronavirus disease 2019 (COVID-19) outbreak has spread throughout the world^1–8^. Currently (September 16, 2020), the cumulative number of confirmed cases worldwide is 29,514,196, and the cumulative number of deaths is 933,806^9^. Most COVID-19 patients have mild to moderate respiratory symptoms^10–16^; however, 13.8% of COVID-19 patients become critically ill with diverse symptoms, leading to multiple organ failure or even death^17–24^. Recent studies have shown that COVID-19 patients with comorbidities, such as endocrinopathies, cardiac disease, chronic respiratory disease, renal disease, chronic neurological disease, and cancer, are more likely to have a relatively unfavorable prognosis^25–35^.

Cancer is a major public health problem that seriously threatens the health of the global population^36^. According to the Global Cancer Observatory, it was estimated that there will be 1.8 million novel cancer cases and 606,000 new cancer-associated deaths worldwide in 2020^37^. Recent studies have demonstrated that cancer enhances susceptibility to COVID-19 and is a risk factor for worse clinical outcomes among patients with COVID-19^38–46^. Liang et al.^47^ reported a cancer prevalence of 1.13% [95% confidence interval (CI): 0.61%–1.65%] among 1,590 cases of COVID-19 in China, which was higher than the overall cancer incidence of 0.29% in the Chinese population. In addition, Giannakoulis et al.^40^ reported a meta-analysis of the outcomes of 46,499 COVID-19 patients with malignancies and showed that all-cause mortality was higher in patients with cancer *vs.* those without cancer [risk ratio (RR): 1.66, 95% CI: 1.33–2.07, *P* < 0.0001]. However, existing studies have been limited to a relatively small sample size, and the incidence of cancer in COVID-19 patients should be further investigated. It is therefore necessary to investigate the relationship between cancer and COVID-19 based on a larger sample size. In this study, we conducted a meta-analysis that included 63,019 participants in 19 clinical studies across 9 countries (China, USA, UK, Italy, Switzerland, Republic of Korea, Iran, Spain, and Portugal) to determine both the incidence and outcome of COVID-19 patients with malignancies.

## Materials and methods

### Search strategy

In this study, we systematically searched PubMed, Embase, Cochrane, Web of Science, and medRxiv databases on July 9, 2020. The search terms included: “COVID-19,” “2019-nCoV,” “SARS-CoV-2,” “clinical characteristics,” “cancer,” “comorbidities,” “malignancy,” “mortality,” “morbidity,” and “outcomes.” The retrieved studies were downloaded from the databases and imported into EndNote X9 for data management and analysis.

### Eligibility criteria

We included studies that met the following criteria: (1) patients studied were confirmed with COVID-19 through clinical and laboratory diagnoses; (2) the study contained information about the number of cases or deaths of cancer and noncancer patients in the population infected with COVID-19; and (3) the language was limited to English. We excluded studies that did not meet our criteria. The exclusion criteria were as follows: (1) articles categorized as reviews, case reports, conference abstracts, or basic experimental research literature; (2) articles that did not include an epidemiological analysis related to the observation indicators of this study; (3) articles that did not obtain complete data or were not a full-text study, and thus, the effective evaluation of the study quality could not be effectively evaluated; and (4) repetitive data published in the literature. To avoid duplication of the sample population, among the studies with overlapping data, we chose the study with the largest sample size among studies with overlapping data. Evaluation of the eligibility of the studies was performed by two authors (Ludi Yang and Peiwei Chai) independently of each other, and conflicts were resolved through consultation with a third review author (Jie Yu).

### Data extraction and quality assessment

Two authors performed data extraction and quality assessment independently of one another. The extraction content included (1) study information: first author, type of study, study period, region of study, source of sample, and total population of the study; (2) population characteristics: age, sex, comorbidities, the number of cancer, and noncancer patients; and (3) outcomes: survival status of cancer and noncancer patients. The data were cross-checked by 2 authors using a standard electronic sheet to reach a consensus. We chose to use the Newcastle-Ottawa quality assessment scale (NOS) to evaluate the quality of the included articles^48^. Articles with ≥ 6 stars were defined as high quality articles, with a total score of 9 stars^49^. Throughout the entire process, if the 2 authors had conflicts or were uncertain, they would consult with the third author to resolve the issue.

### Statistical analysis

We performed statistical analyses using the R META package in R Studio (version 3.6.2). Incidence data were first converted to conform to a normal distribution. Then, a meta-analysis of the conversion rate was conducted to calculate the pooled rate and its 95% CI. For binary variables, the overall effect of cancer on mortality was estimated by the pooled RR with a 95% CI using a random-effects model. *I*^2^ was calculated to assess heterogeneity, and the interpretations were as follows: unimportant, 0%–40%; moderate heterogeneity, 30%–60%; substantial heterogeneity, 50%–90%; and considerable heterogeneity, 75%–100%^50^. We conducted an assessment of publication bias to avoid excessively exaggerating the strength of the association between outcomes and risk factors. Significant heterogeneity was dissected *via* subgroup analysis and sensitivity analysis. A meta-regression was performed to illustrate the potential source of heterogeneity between studies.

## Results

### Data collection

**Figure 1:**
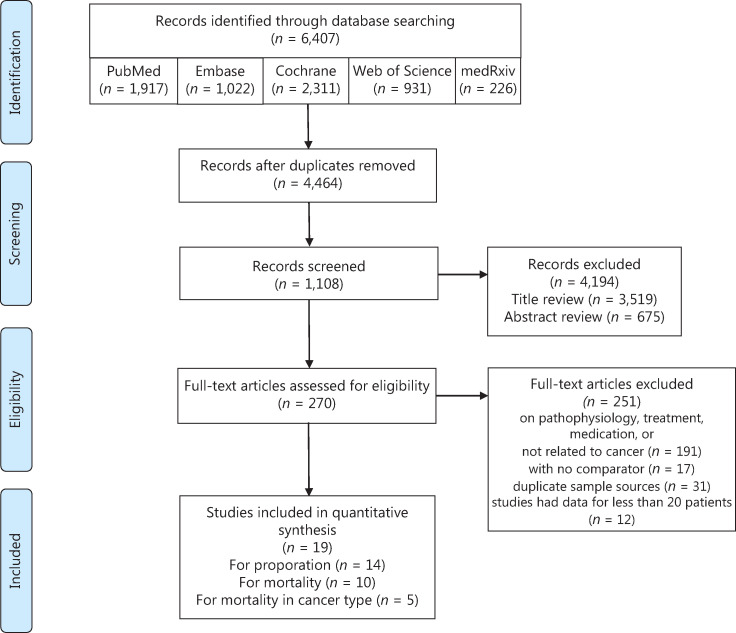
Flow chart of the search process.

In this study, 6,407 articles were retrieved, and 19 articles met the eligibility criteria, with 6 in Asia, 8 in North America, and 5 in Europe. A total of 63,019 patients were included (**Figure 1**), of which 2,682 were cancer patients. For studies with repeated sample sources, we only selected the study with the largest number of samples to avoid duplication of the population. The major characteristics of the included studies are summarized in **Table 1**. The overall quality of these studies was high, with quality scores ranging between 5–9. Assessment of the risk of bias in the involved studies is shown in **Supplementary Table S1**.

**Table 1:** Characteristics of the included studies

Author	Country	Type of study	Total patients	Sex (male)	Median age	Cancer		Non-cancer	
						Total	Dead	Total	Dead
Chinese CDC^51^	China	Retrospective, multicenter cohort	20,812	NA	NA	107	6	20,705	498
Miyashita et al.^52^	USA	Retrospective, multicenter cohort	5,688	NA	NA	334	37	5,354	518
Goyal et al.^53^	USA	Retrospective, multicenter cohort	393	238	62.2	23	NA	370	NA
Baker et al.^54^	UK	Retrospective, single-center cohort	316	173	75	33	10	283	71
Benelli et al.^55^	Italy	Retrospective, single-center cohort	411	359	70.5	33	9	378	63
Rossi et al.^56^	Italy	Retrospective, multicenter cohort	2,653	1,328	63.2	301	44	2,352	173
Nikpouraghdam et al.^57^	Iran	Retrospective, single-center cohort	2,964	1,955	56	17	1	2,947	238
Borobia et al.^58^	Spain	Retrospective, single-center cohort	2,226	1,074	61	385	139	1,841	321
Vasco et al.^59^	Portugal	Retrospective, multicenter cohort	20,270	8,370	NA	603	47	19,667	455
Duanmu et al.^60^	USA	Retrospective, single-center cohort	100	56	45	3	NA	97	NA
Gold et al.^61^	USA	Retrospective, multicenter cohort	305	151	60	12	NA	293	NA
Sami et al.^62^	Iran	Retrospective, single-center cohort	490	299	56.6	15	NA	475	NA
Regina et al.^63^	Swiss	Retrospective, single-center cohort	200	120	70	26	NA	174	NA
Ji et al.^64^	Korea	Retrospective, multicenter cohort	5,172	2,289	42	364	NA	4,808	NA
Joharatnam-Hogan et al.^65^	UK	Retrospective, multicenter cohort	52	31	NA	26	6	26	6
Stroppa et al.^66^	Italy	Retrospective, single-center cohort	56	NA	NA	25	9	31	5
Dai et al.^41^	China	Retrospective, multicenter cohort	641	302	NA	105	12	536	NA
Mehta et al.^67^	USA	Retrospective, single-center cohort	218	127	NA	218	61	0	0
Yang et al.^68^	China	Retrospective, single-center cohort	52	28	63	52	11	0	0
Total			63,019	16,900	—	2,682	392	60,337	2,348

NA, not available.

### The incidence of cancer in COVID-19 patients

The data for cancer incidence in COVID-19 patients was provided by 14 studies (62,000 total patients, 2,256 with cancer). The incidence varied between different countries, with the highest incidence in Spain (17.296%, 385/2,226) and the lowest in China (0.514%, 107/20,812) (**Supplementary Figure S1**).

**Figure 2:**
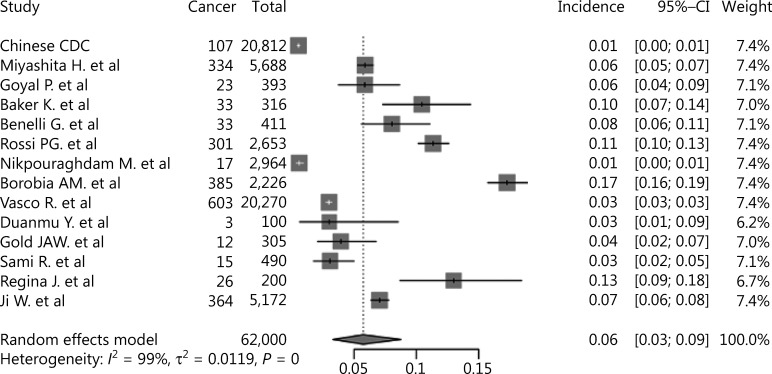
Forest plot showing the incidence of cancer in COVID-19 patients.

**Figure 3:**
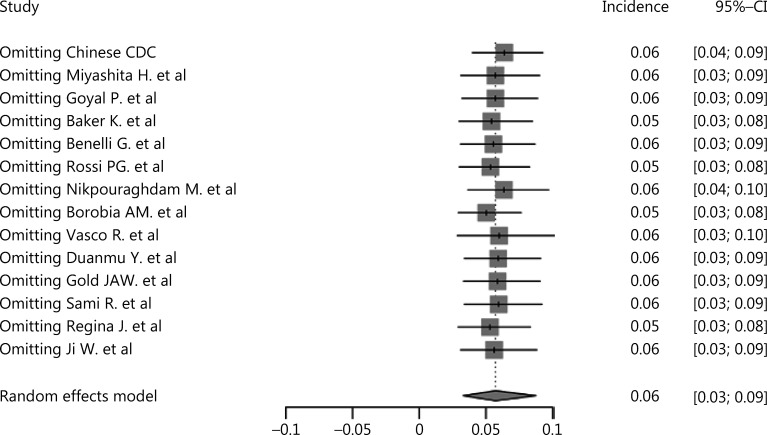
Sensitivity analysis of cancer incidence in COVID-19 patients.

As shown in **Figure 2**, the pooled incidence of cancer in COVID-19 patients was 6% (95% CI: 3%–9%), which was much higher than the global cancer incidence (approximately 0.2%)^69^. There was no significant publication bias in our study (*P* = 0.09). However, our analysis showed that heterogeneity was considerable among the studies (*I*^2^ = 99%). Sensitivity analysis revealed that our results were robust, and the results did not vary significantly after separately omitting each study (**Figure 3**). Subgroup analyses based on the region and sample size were conducted. However, neither could address the source of heterogeneity (**Supplementary Figures S2 and S3**). Using meta-regression, we detected that sex was not the source of heterogeneity (*P* = 0.50, *R*^2^ = 0.00%). Due to the limitation of information, we could not further determine the potential source of heterogeneity.

### Cancer-associated mortality in COVID-19 patients

For analysis of the mortality of the cancer patients infected with COVID-19, 10 studies were included. The total population was 55,448, of which 1,864 were cancer patients. As shown in **Supplementary Figure S4**, the mortality of cancer patients with COVID-19 varied between different countries, with the highest in Spain (36.1%, 139/385) and the lowest in China (5.6%, 6/107).

**Figure 4:**
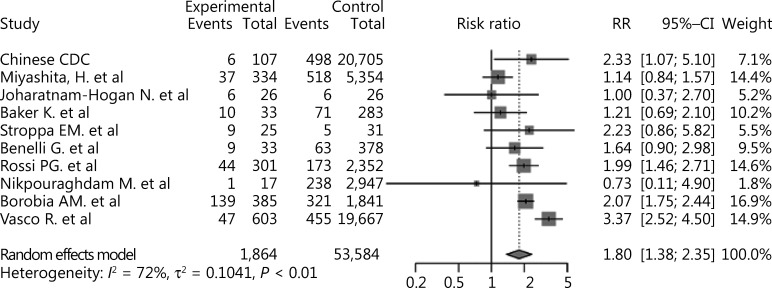
Forest plot showing the mortality of cancer patients with COVID-19.

**Figure 5:**
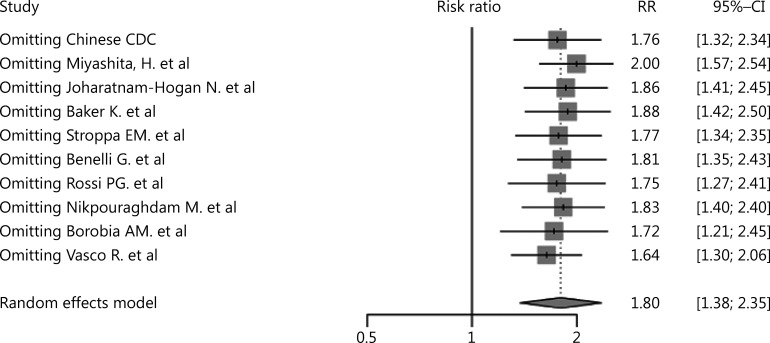
Sensitivity analysis of cancer mortality in COVID-19 patients.

The results showed that cancer was a risk factor for mortality among COVID-19 patients (RR: = 1.80, 95% CI: 1.38–2.35, *P* < 0.01, **Figure 4**). Significant publication bias was not detected among the studies included (*P* = 0.36). There was substantial heterogeneity (*I*^2^ = 72%) in this study, which was studied using subgroup analyses based upon the region and sample sizes. However, inspection of the forest plots of the subgroup analyses built on region and sample size did not reveal the source of the heterogeneity (**Supplementary Figures S5 and S6**). Using sensitivity analysis, we did not observe any obvious change in the results after omission of each of these studies (**Figure 5**). We also performed a meta-regression analysis on sex and found that it was the source of the heterogeneity (*P* < 0.01, *R*^2^ = 96.93%, **Supplementary Figure S7**).

**Figure 6:**
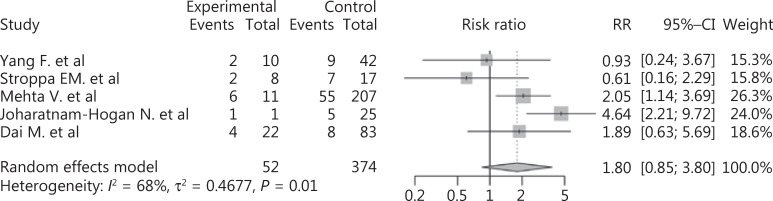
Forest plot showing the mortality of lung cancer patients *vs*. non-lung cancer patients among the COVID-19 population.

We also included 4 studies to compare the mortalities of lung cancer and non-lung cancer patients among cancer patients with COVID-19. The results showed that lung cancer patients were at higher risk of death than non-lung cancer patients (RR: 1.80, 95% CI: 0.85–3.80, *P* = 0.02, **Figure 6**). We further analyzed the effects of COVID-19 on non-lung cancer patients, which indicated that non-lung cancer also increased the mortality of COVID-19 patients (RR: 1.96, 95% CI: 1.57–2.45, *P* < 0.01, **Supplementary Figure S8**). Due to the small number of included studies, no subgroup or sensitivity analysis was performed.

## Discussion

With changes in the living environment and lifestyle, the incidence of cancer is increasing worldwide. According to the Global Cancer Observatory, it is estimated that there will be 1.8 million novel cancer cases and 606,000 new cancer-associated deaths worldwide in 2020^37^. With the outbreak of COVID-19, cancer patients have been affected. A recent study reported that COVID-19 infection of host cells is facilitated by transmembrane protease serine 2 (TMPRSS2), angiotensin-converting enzyme 2, and other host cell proteases, such as cathepsin L, which is highly expressed in cancer patients^15,70–74^. Compared with the general population, the immunosuppressive states of cancer patients make them more vulnerable to severe complications, which may affect the prognosis of the disease^41,52,75^. Apart from the immunosuppressive state, the mean age of cancer patients is greater than the general population, which could be another risk factor for severe COVID-19^47,75^. Several studies have reported that cancer is a risk factor for COVID-19 patients, which could lead to unfavorable clinical outcomes^43,76^. However, a COVID-19 case report from Switzerland detailed a breast cancer patient with immunosuppression who recovered faster and had a better prognosis than her husband, who was in good health^77^. This report indicates that immunosuppression may not always cause severe complications and could even provide advantages in preventing cytokine storms. In addition, Barlesi et al. reported that the death rates of COVID-19 did not differ significantly between the population with and without cancer because of the low percentage of treatment-related adverse events (5.5%)^75,78^. Another study reported that the percentages of severe events in breast cancer patients with COVID-19 were the same as the general population, which might be related to the implementation of much stricter social distancing procedures by cancer patients^79^. Therefore, it is necessary to conduct a comprehensive meta-analysis to identify the relationship between cancer and COVID-19.

This study included 19 high quality articles and we systematically analyzed 63,019 COVID-19 patients worldwide using a meta-analysis. We estimated the incidence of cancer among the COVID-19 population (6%, 95% CI: 3%–9%) and found that it was much higher than that in the general population (approximately 0.2%^69^). Cancer also appeared to be a risk factor for mortality in COVID-19 patients (RR: 1.80, 95% CI: 1.38–2.35, *P* < 0.01), and the mortality was higher in patients with lung cancer *vs.* those without lung cancer (RR: 1.80, 95% CI: 0.85–3.80, *P* = 0.01). A meta-regression was also performed on mortality and found that sex was the source of heterogeneity, which could be related to different sex compositions among different countries^80^. In terms of the susceptibility of cancer patients, the risk of infection was related to the physique of each individual. For example, differentially expressed genes in cancer patients could facilitate the entry of viruses into cells, and dysfunction of the immune system of cancer patients could lead to weaker resistance to viruses. In addition, cancer patients need to go to the hospital for treatment or follow-up regularly, which increases the risk of COVID-19 infection.

Unlike previous studies, our study included a large number of samples, covering wide geographic regions. In terms of data extraction, we expanded the sample size as much as possible. However, for articles with duplicate samples, we only included studies with the largest sample size to avoid duplication of sample sources. The quality of the included studies was assessed using the Newcastle-Ottawa quality assessment scale. Studies with Newcastle-Ottawa scores ≥ 6 were considered to be of high quality. We used the method of sequentially eliminating each study for sensitivity analysis, with the results not changing significantly, indicating that the results of this study were stable and highly representative.

There were also some limitations to our study. First, due to the exclusion of the studies with duplicate samples, the number of included studies was relatively limited. Second, the data used in meta-analysis were from hospital-based studies, which may have deviated from data in the real world. Therefore, some COVID-19 patients with cancer who have not been admitted to the hospital may be ignored. Third, there was a certain degree of heterogeneity in our research. There was also substantial heterogeneity in the incidence study analysis. However, we performed subgroup analysis, sensitivity analysis, and meta-regression, but these analyses could not explain the source of the heterogeneity. We assumed that there were other potential sources of heterogeneity which were not reported. For example, the sample sources involved different regions and different countries where health concepts and lifestyles could have influenced the susceptibility to cancer or COVID-19. Furthermore, the management of COVID-19 and the strategies used to control and prevent COVID-19 differed among countries. These could all be potential sources of the heterogeneity that affected the results.

## Conclusions

In conclusion, cancer was a risk factor for COVID-19 patients, especially lung cancer. The main advantage of our research was that the sample size was large and representative, covering a wide range of regions. This study emphasized the importance of management of patients with comorbidities during the epidemic. Our results could provide useful information as references to protect people at risk of COVID-19. For example, considering the susceptibility of cancer patients, the follow-up interval and the frequency of radiotherapy and chemotherapy during the epidemic could be appropriately delayed to reduce the risk of nosocomial infection. Medical staff must be vigilant for cancer patients in the COVID-19 population, and personalized treatment plans should be developed to prevent the deterioration of the disease.
