# Supplementary material for: Effects of cancer on patients with COVID-19: a systematic review and meta-analysis of 63,019 participants
Source: Cancer Biol Med. 2021 Feb 15;18(1):298–307. doi: 10.20892/j.issn.2095-3941.2020.0559 (PMC7877167; doi:10.20892/j.issn.2095-3941.2020.0559)
Supplement: Supplementary file 1 [file cbm-18-298-s001.pdf]

Supplementary materials

Table S1 Quality assessment of the included studies

Author	Country	Selection	Comparability	Outcome	Total
Chinese CDC[51]	China	***	**	***	********
Miyashita et al.[52]	USA	****	*	**	*******
Goyal et al.[53]	USA	****	**	**	********
Baker et al.[54]	UK	****	**	**	********
Benelli et al.[55]	Italy	****	**	***	*********
Rossi et al.[56]	Italy	****	**	**	********
Nikpouraghdam et al.[57]	Iran	****	**	**	********
Borobia et al.[58]	Spain	****	**	***	*********
Vasco et al.[59]	Portugal	****	**	***	*********
Duanmu et al.[60]	USA	****	**	***	*********
Gold et al.[61]	USA	***	**	***	********
Sami et al.[62]	Iran	****	**	***	*********
Regina et al.[63]	Swiss	****	**	***	*********
Ji et al.[64]	Korea	****	**	***	*********
Joharatnam-Hogan et al.[65]	UK	***	*	**	******
Stroppa et al.[66]	Italy	**	*	***	******
Dai et al.[41]	China	***	**	***	********
Mehta et al.[67]	USA	****	**	**	********
Yang et al.[68]	China	***	**	**	*******

The maximal score of NOS is 9 stars: 4 stars for the selection process, 2 stars for comparability and 3 stars for outcome, with 6 stars or more indicating high quality.

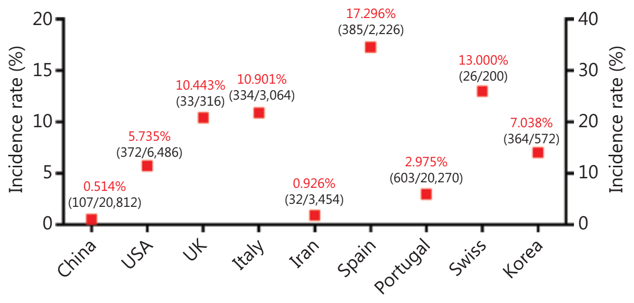

Figure S1 Incidence of cancer in COVID-19 patients among different countries.

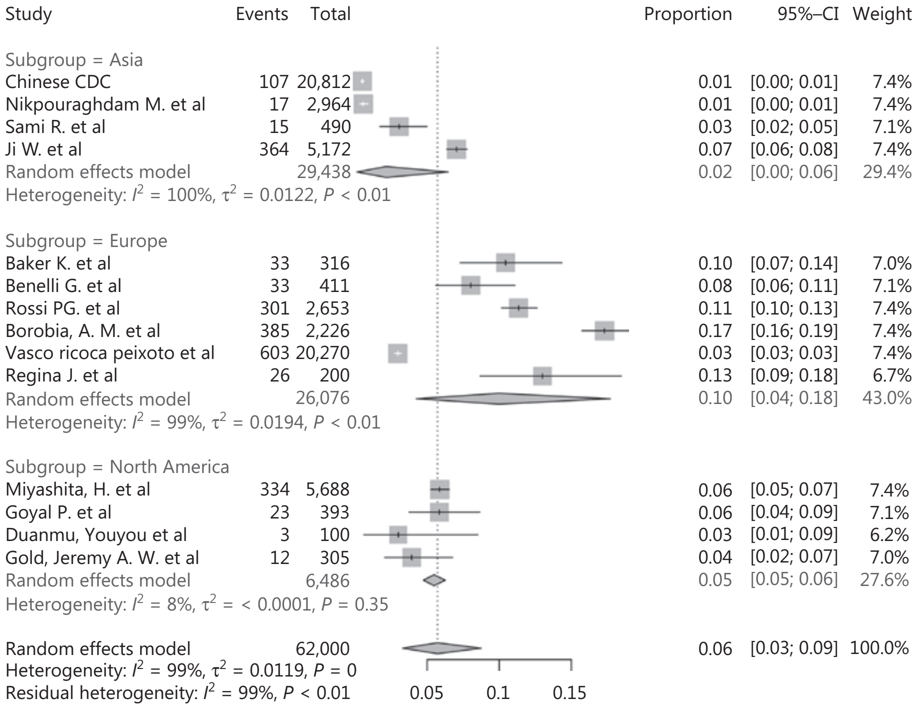

Figure S2 Forest plot for region subgroup analysis of the cancer incidence in COVID-19 patients.

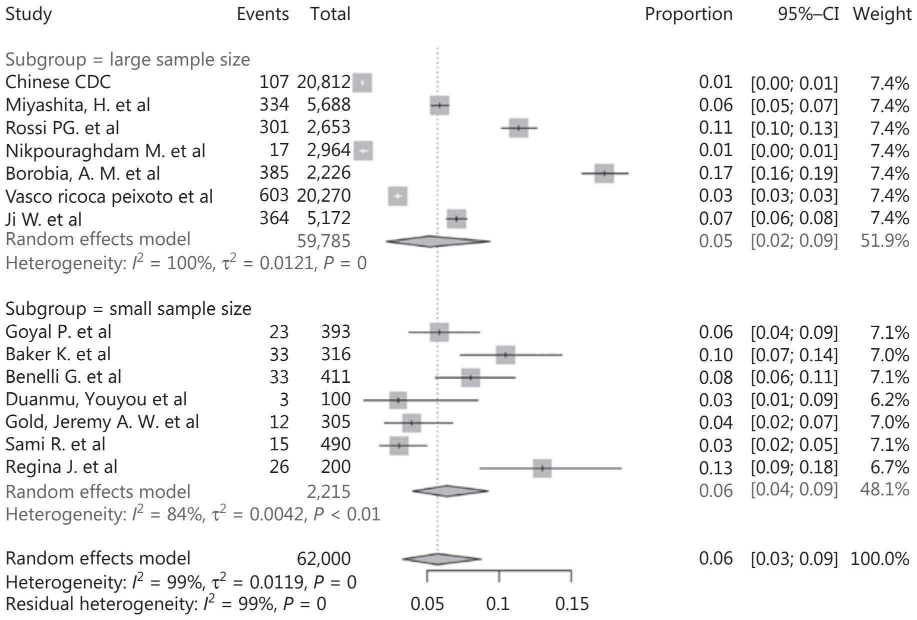

Figure S3 Forest plot for sample subgroup analysis of the cancer mortality in COVID-19 patients.

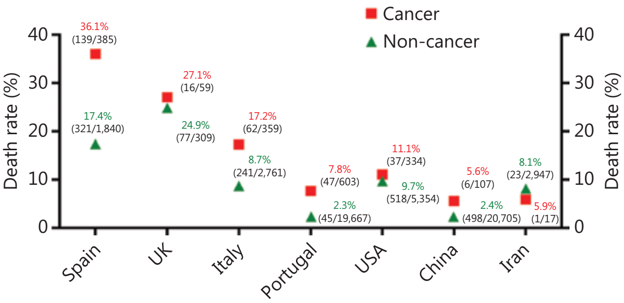

Figure S4 Comparison of mortalities between cancer and noncancer patients among patients with COVID-19 in different countries.

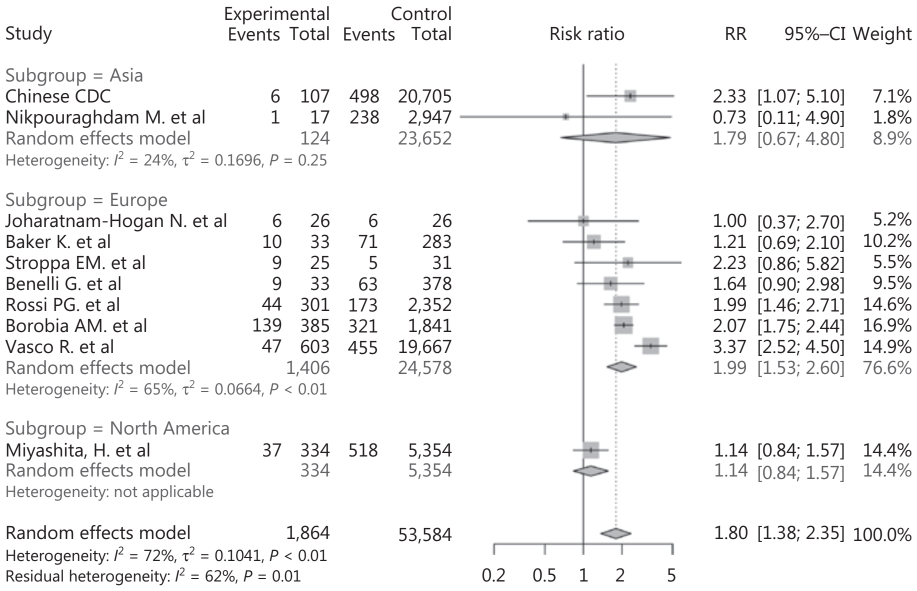

Figure S5 Forest plot for region subgroup analysis of the cancer mortality in COVID-19 patients.

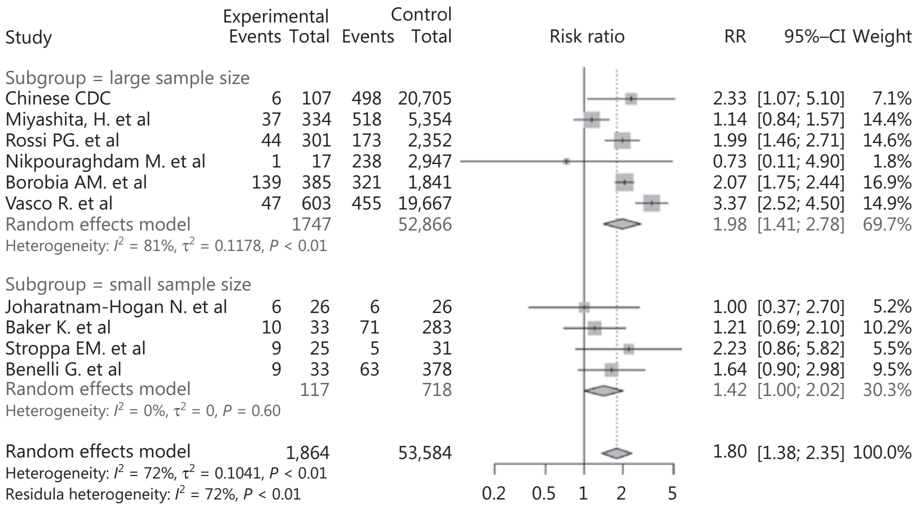

Figure S6 Forest plot for sample size subgroup analysis of the cancer mortality in COVID-19 patients.

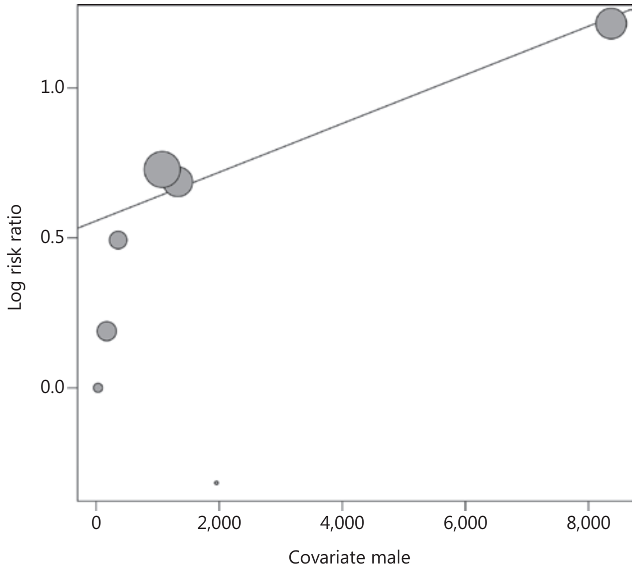

Figure S7 Meta-regression between sex and cancer mortality in COVID-19 patients.

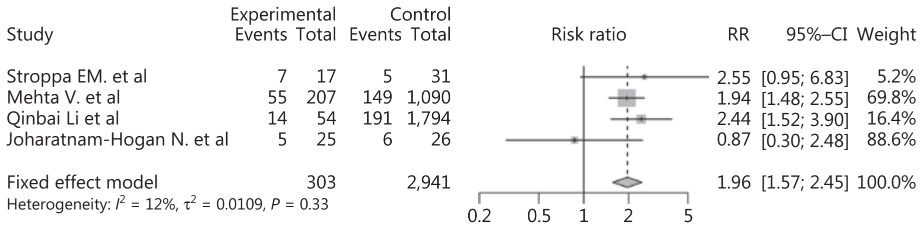

Figure S8 Forest plot showing the mortality of non-lung cancer patients *vs*. non-cancer patients in the COVID-19 population.
